# eHealth Assistant AI Chatbot Using a Large Language Model to Provide Personalized Answers through Secure Decentralized Communication

**DOI:** 10.3390/s24186140

**Published:** 2024-09-23

**Authors:** Iuliu Alexandru Pap, Stefan Oniga

**Affiliations:** 1 Department of Electric, Electronic and Computer Engineering, Technical University of Cluj-Napoca, North University Center of Baia Mare, 430083 Baia Mare, Romania; 2 Department of IT Systems and Networks, Faculty of Informatics, University of Debrecen, 4032 Debrecen, Hungary

**Keywords:** eHealth, mHealth, telehealth, telemedicine, remote patient monitoring, Internet of Things, artificial intelligence, large language model, Matrix open communication protocol

## Abstract

In this paper, we present the implementation of an artificial intelligence health assistant designed to complement a previously built eHealth data acquisition system for helping both patients and medical staff. The assistant allows users to query medical information in a smarter, more natural way, respecting patient privacy and using secure communications through a chat style interface based on the Matrix decentralized open protocol. Assistant responses are constructed locally by an interchangeable large language model (LLM) that can form rich and complete answers like most human medical staff would. Restricted access to patient information and other related resources is provided to the LLM through various methods for it to be able to respond correctly based on specific patient data. The Matrix protocol allows deployments to be run in an open federation; hence, the system can be easily scaled.

## 1. Introduction

In a post-pandemic scenario, the effort of relieving some of the stress that the entire healthcare system had to cope with has led to a growing amount of research regarding implementations incorporating artificial intelligence. As one of the technologies that has a significant impact on multiple sectors, we believe that this proof-of-concept implementation could help facilitate the first steps needed to advance similar healthcare systems towards better performance, fluid human–machine interaction, improved patient support, actual doctor–patient time gains, and reduced costs.

The scope of this paper is to propose an architecture for a modular system capable of interconnecting existing cross-platform chat applications with AI large language models and previously recorded physiological or medical patient data. This study focuses on designing a suitable system architecture that can accomplish the scope and implementing an initial proof of concept to demonstrate its technical feasibility. With this work, we do not intend to improve one specific field of eHealth but to provide a foundation architecture on which specialized architectures can be built.

The two main objectives of our study are the following:

1. Ensure secure communications for the entire system;
2. Provide an AI chatbot with user-specific data isolation.

To accomplish our first objective, we utilize a modern, encrypted, and decentralized communication platform for all types of messaging between medical staff, the AI chatbot, and patients that also allows users to send or receive prescriptions, medical information, instructions, or any type of messages (text, image, audio, video, or file).

The second objective consists of implementing an eHealth AI chatbot that can be accessed only through the aforementioned communication platform that safely allows patients to ask the chatbot any question, even about their own physiological data, prescriptions, pharmaceutical instructions, etc. To achieve this, we customize the context of each question with specific user information so the chatbot will provide helpful and personalized answers based on the actual patient’s data, and if there is not enough information, it will answer accordingly, without misleading the user.

The decision of complementing our previously built eHealth system [1,2] with an artificial intelligence large language model capable of delivering unparalleled functionality through a secure messaging protocol was made after considering all the pros and cons of mixing such technologies with healthcare applications, as reviewed in our previous work [3]. We decided that our current work should be entirely independent of our previously built eHealth data acquisition system so that by using the same method, we could adapt our eHealth AI chatbot to any data source.

### 1.1. Related Work and Challenges

Through the following paragraphs, we present the most relevant eHealth implementation scenarios we found in the works we studied, highlighting some research gaps, pros, and cons, to clarify our motivations for the current work.

We will focus on the following research gaps:

1. Works relevant to ours are based on ChatGPT, which is a paid third-party AI service; hence, it can increase costs and decrease medical information privacy;
2. Security of communications between users and AI is controlled by third parties;
3. We were unable to find any eHealth AI chatbots that use a mature, open protocol to secure communications;
4. There is no seamless communication between patients, medical staff, and AI;
5. There are no local AI chatbots that use Retrieval-Augmented Generation based on the patient’s medical information to offer relevant answers;
6. AI chatbots used in eHealth applications do not restrict the AI to a user-specific context, hence delivering more imprecise or wrong answers;
7. There are no interchangeable AI open model implementations in eHealth;
8. Very few eHealth LLM applications use exclusively open-source software.

Bringing AI technologies into healthcare can be undertaken for several reasons, among which the review in [4] presents the following:

1. Medical notes: medical professionals can use ChatGPT in writing medical records, clinical notes, and related reports;
2. Education: ChatGPT can give medical professionals and students access to additional information and resources;
3. Medical consultation: initial medical consultations, patient information and test results can be summarized to help physicians;
4. Patient triage: ChatGPT can help with patient triage by asking patients about their symptoms and previous medical conditions, attempting to determine the priority of their medical condition;
5. Virtual assistants: with rising telemedicine popularity, creating a virtual assistant that can help patients with making appointments, administering treatment, and handling health records, all from the comfort and safety of their homes, can be very appealing in distant locations;
6. Clinical use: this includes augmenting medical robots, offering dietary advice, or even explaining the dangers behind smoking and other harmful habits.

In trying to build a comprehensive eHealth AI application, we explored related works and searched for solutions to the problems most eHealth applications face. From reviews such as [5], before the launch of ChatGPT, we can observe the variety of natural language processing (NLP) approaches, the difficulties of building applications with natural language understanding (NLU), and how performant pre-trained artificial intelligence language models are compared to other embedding algorithms while taking into account their large size and the expensive training costs. Health literacy, remote assistance, and triage are some of the aspects that can be improved when adopting ChatGPT in low- to medium-income countries (LMICs), but the process needs to take into account several factors like cultural, ethical, and illiteracy rates [6]. We believe that employing a third-party service in the healthcare system of LMICs can pose additional concerns like compromising privacy, liability, and ethics for the sake of having access to AI services. These challenges made us consider implementing remote model access and finding suitable pre-trained models to reduce running cost and increase adoption rate.

One of the first studies [7] that examined ChatGPT’s ability to provide answers to disease-specific questions, evaluating the correctness and the reproducibility of its replies to frequently asked questions, concluded that ChatGPT was able to identify the patient’s emotional response, offered correct first steps for newly diagnosed patients and proactive measures for treatment, and recommended caregivers to help patients find support groups while underlining the importance of mental and physical health for both patients and caregivers. The study [7] excluded user-specific questions and only focused on general answers, which is why we aim to provide the means of asking user-specific questions through our architecture’s implementation.

A study of 54 teleconsultants in healthcare [8] examined how their operations and services were affected by the use of ChatGPT, with positive aspects including increased efficiency, reduction of cost, and diagnostic assistance, while some of the negative impacts included misdiagnosis, reduced medical context and knowledge, and security and privacy concerns related to the fact that sensitive medical information needed to be sent to ChatGPT. Sending sensitive medical information to an online third-party service would raise many concerns, so self-hosting the LLM would allow us to benefit from ChatGPT-like services that bring increasing advantages to healthcare implementations and have their place in future healthcare applications, even if currently, they cannot surpass human execution for certain tasks, as noted in [9]. Pharmacy-related questions can imply greater complexity, and according to [10], even if ChatGPT showed a 65% and a remarkable 85% performance rate for certain categories, it failed with 30–35% rates for drug interaction and dosage categories, highlighting the much-needed improvements the model requires. Additionally, in the world of telepharmacy, both ChatGPT 3.5 and 4 show great performance when asked to act as a pharmacist, offering responses that were relatively safe [11]. Public health dentistry is another field where ChatGPT can have beneficial effects, but as stated in [12], it cannot replace a dentist because it is not able to assist beyond recommending a diagnosis, so in our implementation we need to steer the model towards stating it is not a healthcare professional and refusing to answer without valid context data. To accomplish this, we needed to find an alternative to ChatGPT that we could have more control over. If we do not take these steps, by integrating large language models into chatbots, users could mistakenly think they are presented with specialist medical information, while in reality, AI products are still unable to provide reliable health-related knowledge [13]. ChatGPT is not designed to admit it does not know the answer. In order to avoid such confusion and spreading misinformation, we need to guide our local LLM to offer responsible answers. The latest version of ChatGPT, GPT-4, proved its potential in extracting relevant information from patients in non-English conversations by scoring on average 72.4% in the Chinese Medical Licensing Examination’s Clinical Knowledge [14]. For some queries, all of the tested chatbots (ChatGPT 3.5, ChatGPT 4, BARD, and Claude 2) provided answers that were too complicated for the general public to understand, and only one (BARD) listed sources for later verification [15]. Another research gap is highlighted by the importance of providing context to AI questions, whether it is age, education level, or medical information. In order to safely utilize ChatGPT or similar large language models, we need to take into account the dangers of spreading misinformation, privacy issues, and the possibility of bias [16]. ChatGPT shows evident language bias, but it manages to perform similarly regardless of the language in which the question was formulated [17]. Since the training of LLMs falls outside the scope of this research, the alternative would be to replace the LLM with a less biased one, which in the case of most eHealth applications is not possible, so our solution to this issue was to use an open-source LLM manager.

Our eHealth AI chatbot could be used for theoretical questions, because when used for medical exams, ChatGPT performed well regardless of the type of question, so medical students can trust it for academic aid; however, it was unable to comprehend questions that contained images, to exhibit critical thinking, or to gain insight [18]. Also in healthcare academia, a meaningful ratio of students do not possess enough information about ChatGPT and have a positive opinion about it; hence, they have the disposition of using it for academic purposes, emphasizing the need to better understand the risks involved in using large language models by both students and educators [19]. While taking into account remarkable accomplishments such as ChatGPT’s responses being often indistinguishable from human ones and its accuracy on public health exams well surpassing human accuracy, we should not ignore the risks this entails for the entire educational process, especially in the context of public health education [20]. Accuracy-wise, there is a considerable difference between the responses that ChatGPT generates and the actual information obtained from official sources, even if from a relevance standpoint they can outperform at times [21]. To prevent the spread of misinformation in situations where patients try to self-diagnose, the LLM should only answer questions related to the data we provide and control.

The main use case for large language models in medicine is the AI assistant tool that is intended to interpret symptoms and offer initial steps for diagnosis, treatment, and management; however, as the medical questions become more complex, the model starts facing more difficulties, so further considerations and development of large language models are important in potentially reducing the risks involved in their adoption and increasing accountability [22]. When creating lists of questions destined to help in prenatal counseling, ChatGPT can be a useful resource, but all the generated information should be checked by experts prior to including them in the training materials [23]. In the case of occupational medicine, ChatGPT’s responses did not reach the accuracy of physicians’ answers but managed to attain the same level of completeness as doctors’ answers only when context was included along with the question, emphasizing the importance of providing additional information for the model through text embeddings [24]. In some situations, as in [14], an intermediary between patient and physician can be beneficial to overcoming some of the physicians’ challenges like consulting large numbers of patients, which limits the time allocated for each patient to a few minutes; therefore, in a real-world implementation, the AI would need access to medical information with regard to data privacy. Hence, our eHealth AI chatbot must provide secure communications to safeguard sensitive patient information and potentially fix the lack of interoperability we see between eHealth applications.

Among the most problematic challenges encountered in implementing AI applications is the matter of finding a balance between technological advancement and ethics, with their multiple ramifications (legal, humanistic, algorithmic, and information ethics). Some of the most challenging parts in the development and implementation of ChatGPT in healthcare are as follows [25]:

- Following regulations like the Health Insurance Portability and Accountability Act (HIPAA) for the United States of America;
- Evaluating the potential impact of ChatGPT on human relationships as well as communication between medical staff, caregivers, and patients;
- Monitoring how ChatGPT influences empathy and trust in the healthcare setting;
- Identifying the biases in ChatGPT’s language processing;
- Investigating how ChatGPT can increase or reduce systemic inequalities in healthcare;
- Checking the accuracy, reliability, and transparency of the information supplied by ChatGPT;
- Mitigating concerns about data privacy and security when handing sensitive patient information to ChatGPT for processing.

A similar approach to our RAG implementation is achieved in [26] through prompt engineering and augmenting the LLM with additional knowledge, thus creating a Chat-Orthopedist based on ChatGPT that manages to avoid hallucinations but, compared to our solution, does not provide answers based on user-specific medical information. Keeping medical information private is also the focus of [27], where they use ChatGPT to generate synthetic data to avoid sending private medical information to the ChatGPT API, this being one of the reasons we opted to use local LLMs in our architecture’s implementation.

The novelty of our approach is supported by the lack of research regarding the applications of open-source LLM management software in the context of eHealth and is further strengthened by using non-ChatGPT LLMs. Following a modular approach in integrating LLMs in eHealth applications can allow healthcare solutions to seamlessly adapt to newer and better LLMs, as the number models is ever increasing.

In eHealth, the matter of information security is essential, so works like [28] offer a different approach in securing healthcare systems by using blockchain technology to implement smart contracts responsible for managing device access to data. Federated learning, the paradigm based on the distributed learning concept, can also benefit from blockchain security features [29]. Security and privacy are discussed in the context of healthcare through the metaverse [30], where the packaging of patient’s medical information into a Non-Fungible Token (NFT) could improve the sharing of health records. In an attempt to improve cloud security, ref. [31] introduces a new encryption framework that is a combination of four different encryption algorithms. Another encryption method is proposed in [32], where they manage to decrease encryption/decryption times and reduce healthcare costs by using less energy. While these implementations focus on improving medical information security, one of their limitations is the fact that they do not present a clear template on which other researchers could build upon. Many eHealth researchers need to implement their own secure communications because the state of the art lacks architectural templates to support this. Hence, as a contribution for our architecture’s implementation, we avoid building a security system from scratch, so we aim to integrate a tried and tested open secure communication platform.

To conclude our motivations for the current work, we list the most relevant aspects we aim to improve through our proof-of-concept implementation, proving the novelty of our work:

1. Running costs: by providing remote model access to suitable pre-trained models, we avoid spending enormous sums on training models and become independent from third-party online AI services;
2. AI answers: we instruct the LLM to state it is not a healthcare professional and to avoid answering questions without our specific context information, improving its reliability;
3. Replicability: by exclusively using open-source software components, we allow others to freely replicate our solution by simply copying and personalizing it;
4. Scalability: our system’s architecture could be implemented in an entire network of healthcare facilities because the communications platform we use has this functionality built in;
5. Privacy: we avoid sending sensitive medical information to an online third-party service by self-hosting the LLM, so the privacy of the medical information is protected from online third parties;
6. Security: the vast majority of related implementations use ChatGPT or other third-party AI services, entrusting them with the security of their communications. In our solution, we self-host the LLM and utilize a secure open communications protocol to keep everything under our control;
7. Interoperability: by using an open communication protocol, we could allow other eHealth systems to communicate with our entire federation of servers.

### 1.2. Implementation Choices

The avalanche of large language model applications began after the success of ChatGPT, and since its unveiling, numerous businesses and institutions have started experimenting with chatbots. Among these diverse applications, some started experimenting with healthcare implementations. AI applications range from frivolous to vital ones like emergency situations [33,34,35], and with every additional functionality, there are many risk factors that need to be taken into consideration, like inaccuracies, wrong information, large language model hallucinations, and data privacy and security when handling patient data. With these risks in mind, knowing we need our AI model to have direct access to patient data, we determined that hosting and running a large language model locally would eliminate these security and privacy concerns, because this way, no sensitive data ever leave our system, so no third-party service has access to our data.

Self-hosting an open-source large language model addresses the security and privacy concerns regarding third-party services, so to overcome potential data privacy and communication security issues, we decided to use a decentralized open communication protocol called Matrix. After studying the protocol [36,37,38], we researched its adoption, which revealed the following:

1. The chat platform for officials and citizens (Luxchat4Gov) in Luxembourg is based on the Matrix open protocol [39];
2. The French government messaging service Tchap is also based on the Matrix open protocol [40];
3. Germany’s united armed forces (Bundeswehr) started testing a Matrix implementation called BwMessenger [41];
4. Most importantly, the German healthcare sector’s digital solutions provider, Gematik, started rolling out the TI Messenger communications platform in the last few years [42].

The Matrix open protocol offers a plethora of functionalities, and here are some of the advantages it brings to our implementation:

1. Communication encryption support;
2. The communication server can be self-hosted either locally or in a federation of private servers;
3. Multiple server software choices, all open source (Synapse, Dendrite, Conduit, Conduwuit, Construct, etc.);
4. Multiple client applications for desktop, mobile, and web (Element, Element X, FluffyChat, Quadrix, etc.);
5. Support for bridges that can allow users to be notified even on other messaging networks;
6. Advanced user management and access restrictions to conversations, rooms, and resources;
7. Possibility of managing all patient contact through application features like text conversations, voice and video calls, file attachments, and more.

## 2. Materials and Methods

In the following paragraphs, we offer an overview of how all the parts of our system are tied together and how it all works. It consists of multiple components that could be implemented in a variety of ways, meaning that some parts could be hosted on the same machine. Due to the nature of the Matrix protocol, we could scale the system to multiple locations and servers, providing access to our eHealth bot in any scenario.

The main components of our system are the following:

1. IoT eHealth data acquisition system [1] (optional);
2. Matrix server;
3. Large language model;
4. eHealth AI chatbot.

**Figure 1:**
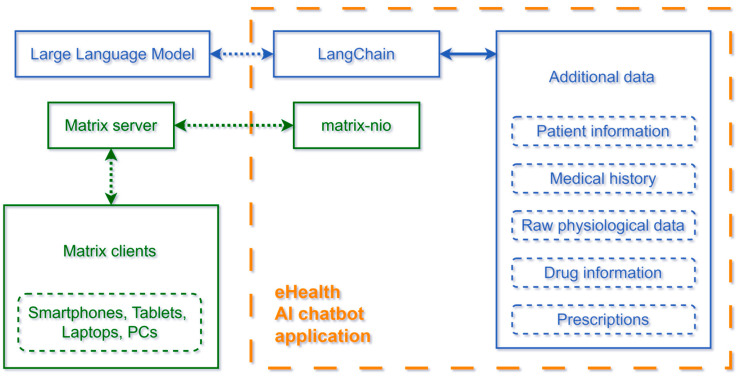
Overview of the entire system.

An overview of the system can be seen in Figure 1, where we can observe the Matrix protocol related communications in green and all the LLM-related communications in blue.

Both the LLM and the Matrix server can run on machines separate from the eHealth AI chatbot application, but this is not a requirement. For fast, almost instant chatbot replies, the LLM must run on a machine that has a modern GPU with enough memory to load the entire model in VRAM, otherwise the performance of the LLM is severely diminished.

Architecturally, we tried to keep the system modular and have a clear data flow, as described below:

1. Matrix communication: All users chat with the system through Matrix clients, which communicate with the eHealth AI chatbot through Matrix servers;
2. LLM communication: The main Python application (eHealth AI chatbot) is the only component with access to sensitive patient information. This component prepares the user’s information and combines it with the user’s message, forming chains of prompts that are sent to the LLM. The LLM’s response is sent back to the user through the same Matrix conversation.

One of the main challenges in implementing this eHealth AI chatbot was the context window, the number of tokens our large language models can process at once (mostly 2048 tokens), especially for the ones we had enough resources to host and run locally.

In our tests, we wanted to analyze how accurate and reliable the answers of our eHealth AI chatbot were, so we extracted relevant physiological data recorded through the eHealth data acquisition system from our previous work. Because of the context window restriction and to better test our chatbot, we carefully selected and anonymized test data samples to avoid any privacy issues and be able to better evaluate the performance of our eHealth AI chatbot, creating a sample single-user test dataset.

Below, we describe each component in detail with all the essential characteristics, configurations, and approaches.

### 2.1. IoT eHealth Data Acquisition System

Our previously implemented physiological data acquisition system allows us to record, store, and stream raw sensor data in real time. We used this system to collect data like blood pressure, temperature, galvanic skin response, and air flow. The eHealth data acquisition system [1] is running on a Raspberry Pi 3 and can be connected directly to the eHealth AI chatbot application. In our test, we use anonymized data samples, but in a production deployment we would connect the chatbot directly to the recorded raw data.

Because our system is modular, the eHealth data acquisition system could be replaced with any other physiological or medical information data source.

### 2.2. Matrix Server

There are a significant number of servers available in the Matrix ecosystem.

Matrix servers [43] come in a variety of development stages (stable, beta, and alpha), licenses, and programming languages, the most important ones being, at the time of writing this paper, as follows:

1. Synapse: Python, stable;
2. Conduwuit: Rust, beta;
3. Construct: C++, beta;
4. Conduit: Rust, beta;
5. Dendrite: Go, beta;
6. Telodendria: C, alpha.

Because of the nature of our eHealth application, needing to take every security and privacy measure into account, we had to have total control over our Matrix server, so we installed and configured the Dendrite server in a Docker container on a computer running Linux. We chose Dendrite because it is still under active development and in our tests, it proved to be stable and needing fewer resources than the main Synapse server.

In a multilocation scenario, we could install several Matrix servers, and they could communicate with each other, this being useful in a nationwide implementation, where each hospital or region could have its own Matrix server to prevent any downtime.

As the Matrix protocol is open and designed with encryption in mind, we can address the issue of secure communications in eHealth by directing all our communications through the Matrix server. Also, by allowing all users (human or AI) to chat through this Matrix platform, we ensure seamless communication between patients, medical staff, and the AI chatbot.

### 2.3. Large Language Model

Having security and privacy restrictions, we could not use services like ChatGPT or any other third-party AI chatbot Application Programming Interfaces (APIs), because we needed to have full control over the handling of sensitive patient data.

We recognize the ethical concerns [44] regarding LLM bias and fairness, so in our modular system architecture, we opted for a plug-and-play approach to LLMs, meaning that by integrating with Ollama [45], we provided the ability to easily switch from one LLM to another, without affecting patient data, Matrix server integrations, or any other settings. While we do not evaluate or mitigate LLM ethical issues, the system administrators could effortlessly switch to another LLM that provides a higher degree of fairness.

There are multiple tools that allow us to run LLMs locally, but one of the most stable ones is Ollama. It serves an API on a specific port, so our chatbot software can connect to it similar to ChatGPT’s API. Ollama supports downloading several models from their repository and using them through its API or Command Line Interface (CLI), this being our solution to the issue of interchangeable AI models. Another side to this decision is the contribution to the state of the art, as there is a shortage of non-ChatGPT eHealth AI applications, especially based on local and open LLMs, which can solve privacy issues and decrease costs.

Context sizes of LLMs can vary, but for our hardware (NVIDIA GeForce RTX 4060 Ti 16GB), most models had a context window of 2048 tokens. With the help of Ollama’s API, we were able to cycle through multiple models, assessing their accuracy, speed, and consistency, determining which LLM was best suited for our chatbot. LLM performance falls outside the scope of this paper, but we had to ensure our eHealth AI chatbot would perform as expected. In our tests, we concluded that the following worked best for our application:

- Llama 3 8B from Meta;
- Phi-3 3.8B from Microsoft;
- Mistral 7B from Mistral AI.

### 2.4. eHealth AI Chatbot

Having such a limited context window, LLMs cannot receive substantial amounts of input along with their prompt. To personalize their response, we had to use a method called Retrieval-Augmented Generation (RAG) [46,47,48], implemented using LangChain, which allows the model to access relevant information in addition to its trained knowledgebase. Along with [47], we can also review the comparison between fine-tuning a model and using the RAG method, as the study highlights RAG’s suitability in healthcare applications. LangChain has been used in other privacy-focused implementations like MedAide [49], where the previous version of Llama3, the Llama2 7B model, was chosen as the most performant.

After we generate embeddings for the additional data, the model accesses and indexes the embeddings with FAISS (Facebook AI Similarity Search from Meta), then allows LangChain to perform similarity searches on the data, or in our case to convert the newly created vector store into a retriever-class object, which can be used in other methods supported by LangChain.

The eHealth AI chatbot software component is written in Python (version 3.11.8) and uses two essential Python packages:

1. LangChain [50]: Can “chain” multiple components together to accomplish complex tasks. LangChain can use multiple vector stores. We use LangChain to populate the vector store, connect to the Ollama API, invoke a response from the LLM, and allow Matrix-nio to transmit it to the specific room or chat it was requested in.
2. Matrix-nio [51]: With the help of this Python package we were able to connect our chatbot software to the Matrix server, verify identity, join rooms, initiate chats, respond to questions asked by users, etc.

Using LangChain, we can create loaders for different types of information, including webpages, documents, or practically any type of information that we can access locally or remotely.

By using RAG with carefully tailored, user-specific information, our solution not only provides relevant AI answers but also eliminates LLM hallucinations and most imprecise answers.

In our tests, we provided the following types of information to the retriever:

1. JSON files: These include measurements for blood pressure, pulse, and weight, patient information (first name, last name, birthdate, patient ID, email, etc.), patient’s medical conditions (condition name, onset date, confirmation date, etc.), and the prescriptions of currently administered treatments;
2. PDF files: To better control the answers about medication, we included entire leaflets of instructions about the drugs the respective patient was taking. For this scenario, we could later implement an online search component that could download these instructions from official databases and cache them for later use;
3. Extra information: We tried to remedy some of the basic flaws of LLMs, like not knowing what date it is. One of our most successful attempts was to always supply the chatbot with additional information, telling it what date and time it was right before starting to generate an answer.

## 3. Results

Since the focus of our work was the design and implementation of an architecture for an eHealth system capable of fulfilling our main objectives, the performance of the chosen communication platform and AI large language models were not the focus of our tests, so we only analyzed the functionality of the extra features we built in our proof-of-concept application.

We managed to implement our main objectives by integrating the tried and tested Matrix protocol and the Ollama project into a novel eHealth AI chatbot application, bridging features from these projects to solve most security, privacy, and financial concerns that prevent similar eHealth applications from being implemented successfully on a larger scale. Here we highlight the most important features of our eHealth AI chatbot system:

1. The Matrix communication platform, LangChain, as well as the Ollama project, are open-source;
2. The Matrix ecosystem already contains several server and client implementations, so the system administrators can choose which ones to use according to their hardware specifications. The freedom of choice also applies to users, allowing them to choose their web, desktop, or mobile application according to the device they are using;
3. The hardware necessary to run the entire system can be limited to one consumer-grade PC, and as the number of users increases, it can be easily scaled, so this implementation can be deployed even in low-income regions;
4. Matrix ensures that all the sensitive information transferred from one user to another is encrypted, thus solving an important eHealth issue;
5. Having a decentralized communication platform can provide additional benefits, like security, privacy, data ownership, resilience, independence, and federation;
6. Patients have access to their medical information, prescriptions, and medicine leaflets through an AI chatbot that is securely hosted and only communicates with its users through the Matrix protocol;
7. The AI chatbot offers personalized health information through a chat interface in a more natural and efficient way. If the requested information is missing, the chatbot will answer accordingly.

In the following paragraphs, we present our results, providing screenshots taken from the Element cross-platform application. The screenshots include question–answer pairs taken directly from the eHealth AI chatbot room, highlighting only the features corresponding to our main objectives, without including the plethora of functionalities inherited from the included software projects (Matrix, Ollama, etc.).

**Figure 2:**
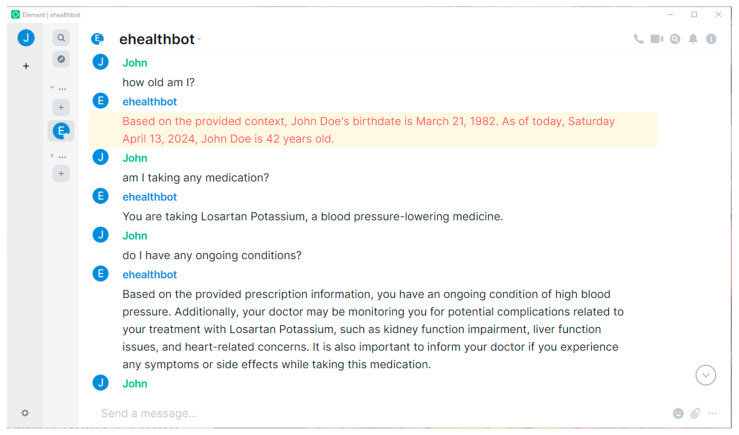
A patient’s conversation with the eHealth AI chatbot showing how the LLM managed to recall the patient’s name, calculate his age, locate treatment information, find ongoing medical conditions, and provide potential medication complications directly from the PDF leaflet.

In Figure 2 we observe a basic interaction, where the eHealth AI chatbot recalls the patient’s name from the context file provided and manages to calculate his age. For the second question, the answer is extracted from another JSON file, “prescription”, where the treatment is described in detail. While the last answer is correct, the model omitted the fact that this patient also suffers from another condition for which he does not have any treatment assigned. We observe how information from a medication leaflet PDF file is intertwined with the patient’s treatment data to form a comprehensive answer.

**Figure 3:**
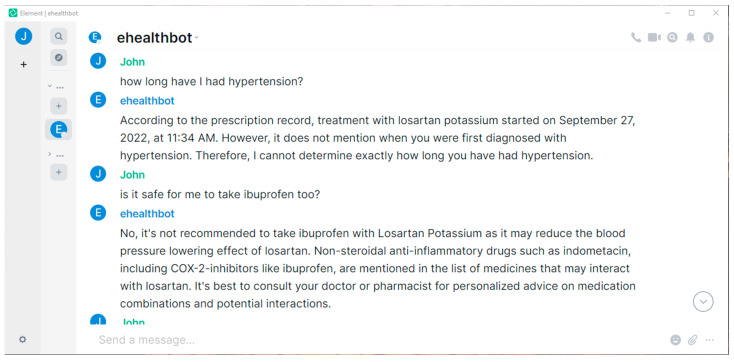
The eHealth AI chatbot managed to find the start of the treatment, failed in finding the first diagnosis, but successfully advised against mixing incompatible medications.

As we see in Figure 3, the eHealth AI chatbot extracted the relevant date about the start of an ongoing treatment, failed in finding the date of the initial diagnosis, but managed to correctly recommend the user against taking another medication that, according to the leaflet, can cause adverse effects when taken along with his current treatment.

**Figure 4:**
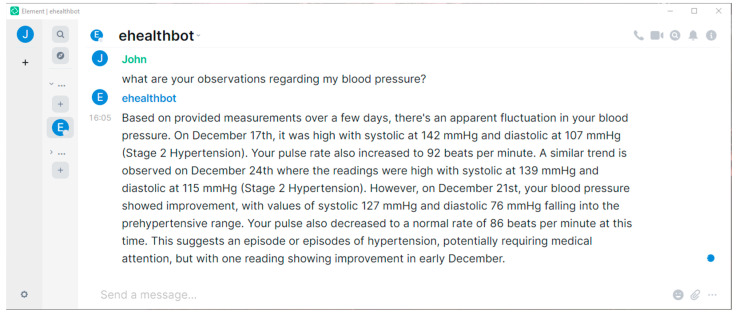
A conversation with the eHealth AI chatbot where it excels in extracting meaningful blood pressure recordings from tens of files and clearly describing its findings.

Figure 4 shows the potential of LLMs, searching tens of files of blood pressure recordings and being able to extract meaningful information while formulating these findings in a syntactically correct manner, close to a human being.

By using open-source software exclusively, we managed to reduce costs to a minimum and ensure a high level of replicability, since our architecture could be copied by others at no additional cost. Of course, we are not considering other use cases, locations, and variables that fall outside the scope of this paper.

In our tests, we found that the very nature of LLMs [52] can be the greatest limitation of our study because the answers can be unpredictable; hence, the reproducibility of the conversation is one of the largest issues. Even though switching LLMs could lead to biased results, we took the decision to design the system this way because the ability to use specific LLMs for certain situations can be extremely useful. The accuracy or performance of LLMs is not within our focus, but we integrated the mechanisms necessary to guide LLMs into providing precise answers based on useful custom data. The first step in stopping the LLM’s hallucinations was to force it, through specific prompts, to only answer if it is provided with the relevant information. Due to the nature of how LLMs work, their responses have great variability, and this leads to unclear or even wrong answers, showing that before adopting such technologies in real-world scenarios, more research is needed.

## 4. Discussion

The authors’ contributions consist of writing the eHealth AI chatbot Python application; preparing anonymized test samples from previous works to avoid privacy issues; configuring, hosting, and testing all the components of the system including the Matrix server and clients and LLM server; and partially adapting the previously built eHealth IoT data acquisition system.

The most important contribution of our research to the state of the art is the architecture itself, ensuring the system has great potential to grow. We believe that most of the works we studied could benefit from implementing our architecture, even partially, by continuing to use ChatGPT or their preferred AI service instead of a local LLM while adopting the rest of our architecture.

By basing all our communications on the Matrix open protocol, we only secure communications between Matrix chat applications (users and medical staff) and the eHealth AI chatbot, but the data acquisition, user management, and server administration should also be secured locally to prevent potential vulnerabilities.

During development, we found that our approach of integrating the eHealth AI chatbot into a well-established communications platform opened the door to a plethora of possibilities, among which are the following:

- Every user could interact with the eHealth AI chatbot, meaning that not only patients but also medical staff could benefit from its features. They could ask questions about one or more of their patients, improving search speed, finding connections between related cases or even asking details about upcoming procedures;
- Communicating through the Matrix platform could increase remote collaboration between doctors by allowing them to discuss problematic cases or by responding to patient questions, all from the same application;
- The AI chatbot could be added to any room, assisting multiple doctors in discussing specific cases;
- The type of communication could fit the needs of different patients or cases. In urgent situations or when the patient has a disability, communicating through voice or video might be preferred;
- Messages could include images, audio, video, and other files, which could save a lot of time for both patients and doctors.

In addition to the above possibilities, our application was able to connect information from multiple different sources and to offer a precise answer that would prevent the user from having adverse reactions caused by adding new medication to the existing treatment. These findings indicate the importance of integrating modern communication methods with AI and eHealth, giving healthcare applications more possibilities to improve our quality of life.

Compared to one of the closest implementations we found, our current research is more focused on providing a secure, encrypted, and versatile communications platform (Matrix) in contrast to the limited user interface (Streamlit) used in [49], and using a different LangChain approach where the model can access user-specific medical data instead of a collection of online databases. Still, allowing our system to access these databases could improve AI answers, especially in situations where it does not have enough information about a certain user.

Future work or potential enhancements based on this proposed foundation could include the following:

- In a production deployment, we could implement multiple answers based on several LLMs, so users could evaluate the answers they receive and provide useful feedback for mitigating LLM ethical issues;
- Following the Matrix protocol model, we could analyze if the eHealth AI chatbot software should be hosted on each eHealth IoT Data Acquisition System or choose a centralized approach;
- To better serve each patient, regardless of which minority they belong to, we could either use multilingual or entirely different language models based on the specific Matrix server’s location. This could be a preference the patient can configure in their account profile;
- Implementing prescription functionality on top of our system would be straightforward because the communication infrastructure supports several data types;
- Disease detection based on computed tomography scans and bloodwork could be implemented by adding specialized classification models trained to detect certain medical conditions. This could prove helpful especially in the situation where doctors must examine huge numbers of patients and the time allotted to each patient is too short. Hence, the highlighting of potential issues doctors need to take into consideration when examining a patient could save lives;
- Access to official medical information repositories could be implemented through the LangChain API, enabling the eHealth AI chatbot to find reliable data when needed.

While navigating all the challenges regarding ethical, privacy, and security concerns, we believe that by curating medical information access, future implementations will also be able to improve disease prevention and treatment. We have come to believe that the proportion and complexity of AI healthcare implementations will continue to rise, so it is of paramount importance to safeguard the rights of patients and to adjust the legal and social frameworks. As the sophistication of such systems increases, so does the potential for vulnerabilities and errors.

## 5. Conclusions

With our proposed architecture, through our proof-of-concept implementation, we manage to provide a comprehensive eHealth system that provides solutions to several problems like privacy, security, and LLM answer relevance and correctness. The proposed system aims to solve some of the most important problems in healthcare applications while providing a more natural conversational interface and maintaining costs at a minimum.

In conclusion, our work delivers a novel approach that can be employed by other eHealth applications and addresses relevant research gaps by showing the plethora of possibilities AI can bring to the healthcare system just by gaining access to the patient’s data and by using modern communication platforms. The results showcase the unrealized potential of providing much improved healthcare services with privacy, security, and convenience in mind.

## Data Availability

Data available on request due to restrictions.
